# Body mass index and partial remission in 119 children with type 1 diabetes—a 6-year observational study

**DOI:** 10.3389/fendo.2023.1257758

**Published:** 2023-09-14

**Authors:** Magdalena Sokołowska-Gadoux, Przemysława Jarosz-Chobot, Joanna Polanska, Alicja Kalemba, Agata Chobot

**Affiliations:** ^1^ Department of Children’s Diabetology and Pediatrics, John Paul II Upper Silesian Child Health Centre, Katowice, Poland; ^2^ Department of Children’s Diabetology and Pediatrics, Medical University of Silesia, Katowice, Poland; ^3^ Department of Data Science and Engineering, Silesian University of Technology, Gliwice, Poland; ^4^ Department of Children’s Diabetology and Pediatrics, School of Medicine in Katowice, Medical University of Silesia, Katowice, Poland; ^5^ Department of Pediatrics, Institute of Medical Sciences, Opole University, Opole, Poland

**Keywords:** partial remission, BMI z-score, type 1 diabetes, obesity, observational study

## Abstract

**Background/objective:**

This long-term study aimed to analyze the associations between BMI *Z*-score, HbA1c, and daily insulin requirement (DIR) and the prevalence and duration of partial remission (PR) in children and adolescents with type 1 diabetes (T1D).

**Methods:**

After retrieving retrospective data for 195 patients from their health records at 24, 48, and 72 months after T1D diagnosis, the study group was comprised of 119 (57 girls) children with a complete dataset for all 6 years. PR was defined according to the ISPAD guidelines. Analyses were carried out in the whole group and subgroups according to PR duration: no PR at all (NPR), PR lasting less than 2 years (PR < 2), and PR at least 2 years (PR ≥ 2).

**Results:**

PR was observed in 63% of the patients (78.9% of overweight and 100% of obese patients). NPR patients showed the lowest mean initial BMI *Z*-score [−0.65 ± 1.29 vs. 0.02 ± 1.42, (PR < 2), *p* = 0.01 and vs. 0.64 ± 1.43 (PR ≥ 2), *p* = 0.17]. The dissimilarity in BMI across patients declined over time. Within the NPR group, the initial mean BMI *Z*-score significantly increased within the first 2 years (unadjusted *p* < 0.001) and remained constant afterward. In the PR <2 group, the highest increase in BMI *Z*-score occurred after 4 years (*p* < 0.001) and then decreased (*p* = 0.04). In the PR ≥2, the BMI *Z*-score slightly decreased within the first 2 years (*p* = 0.02), then increased (*p* = 0.03) and remained unchanged for the last 2 years. Six years after T1D started, the mean DIRs do not differ among the patient groups (ANOVA *p* = 0.272).

**Conclusion:**

During 6 years of follow-up, PR occurred in almost two-thirds of the studied children including almost all overweight and obese children. We observed a gradual normalization of the BMI *Z*-score at the end of the follow-up. BMI *Z*-score increased slightly in children with no remission initially but remained later constant until the end of observation. In both remitter groups, the increase in BMI *Z*-score appeared later when the protective honeymoon period ended. Regardless of BMI *Z*-score, the β-cell destruction process progresses, and after 6 years, the DIR is similar for all patients.

## 1 Introduction

Most often, the symptoms of type 1 diabetes mellitus (T1D), caused by progressive, autoimmune destruction of pancreatic β-cells leading to insulin deficiency, appear when more than 70% of β-cells are damaged (1). This destruction begins several years before the clinical diagnosis of diabetes, but some of these cells can still be functional even decades after the onset (2–4) with detectable C-peptide after as long as 50 years of diabetes (5).

Often, shortly after initiating appropriate insulin therapy, a period called partial remission (PR) or more colloquially “honeymoon phase” can occur. It is characterized by transient recovery of residual β-cells and increased endogenous insulin production due to metabolic improvement reversing the effect of glucose toxicity (6, 7). During this period, it is easier to achieve optimal glycemic control with smaller doses of insulin, and there is a lower risk of severe hypoglycemia (3, 6). The prevalence of PR differs depending on the studied cohort, geographical region, and selected definition. On average, it develops in approximately half of children with newly diagnosed T1D (1, 8), and a few of them (0%–3.2%) may even develop a complete remission (9, 10).

This particular phase—if it occurs—may have important consequences for the patients because of its short- and long-term impact on the course of diabetes, potential chronic complications, and quality of life (3, 8). Patients who experienced PR had a significantly lower risk of microangiopathic complications at 7 years of follow-up (11). Moreover, in a 5-year retrospective cohort study, children and adolescents with T1D who experienced PR had lower LDL cholesterol levels 4–5 years after diagnosis, suggesting the cardiovascular protective effect of PR (8, 12). This is important to remember that cardiovascular diseases have the highest impact on mortality rate in adults above 30 years old with T1D (13). Therefore, immunologic and metabolic factors that may extend this period or even stop the destruction of the remaining β-cells are studied with increasing intensity (1).

This study is a continuation of our former research project (14) in which we retrospectively, across 48 months, evaluated the associations of chosen clinical and laboratory factors in newly diagnosed T1D children with PR. Our results, supported by those of another study (4), pointed out that PR developed more often in children with higher body mass index (BMI) (14) and prompted us to specifically investigate the relationship between BMI and PR.

## 2 Materials and methods

Retrospective data were obtained for 195 patients (100 boys, 95 girls) from 16 months to 18 years old diagnosed with T1D between the years 2012 and 2013 and remained under the care of the largest regional pediatric diabetes center (Upper Silesian Centre of Child’s Health in Katowice) and the Center of Reference of the SWEET (Better control in Pediatric and Adolescent diabeteS: Working to crEate CEnTers of Reference) network (https://www.sweet-project.org/) in Poland. T1D was diagnosed based on the criteria presented in the International Society for Pediatric and Adolescent Diabetes Clinical Practice Consensus Guidelines (ISPAD CPCG) and positivity to anti-islet antibodies (15). Patients with diabetes other than T1D were excluded from the study. All patients were treated according to the Diabetes Poland recommendation, which has not changed over the years (16, 17). The treatments included functional intensive insulin (adapting dose) therapy, initially intravenously and then subcutaneously, using multiple daily injections or a personal insulin pump, with the goal of achieving normoglycemia within 48 h of treatment.

**Figure 1:**
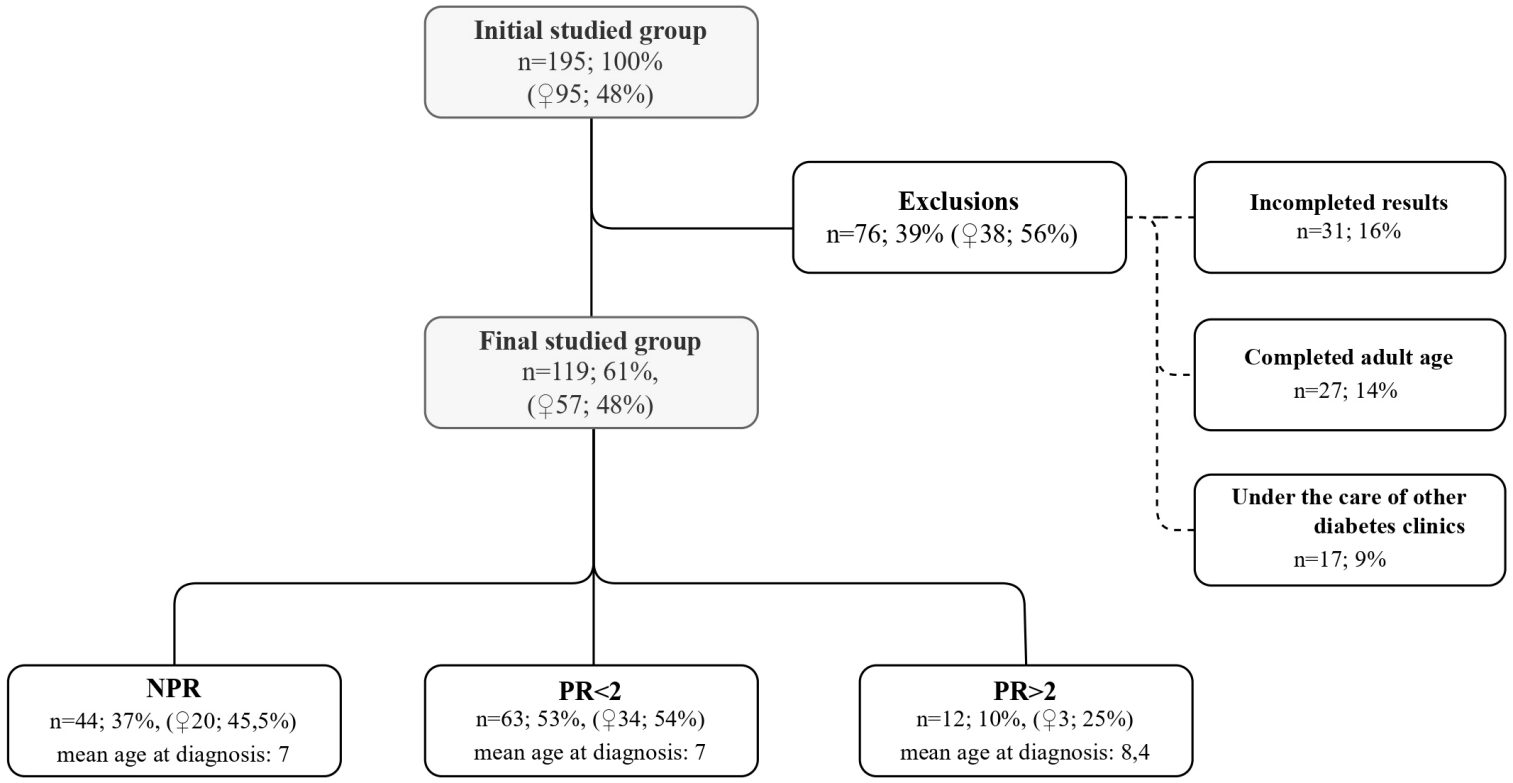
Characteristics of the initial studied group, exclusion criteria and the final group divided into subgroups.

The patients were routinely followed up in our diabetes center at least every 3 months. Data were retrieved from the health records at the following time points: 24, 48, and 72 months after T1D diagnosis or the visit closest to this time point (maximal divergence was 4 to 6 weeks). The study group for statistical analyses was comprised only of patients who had a complete dataset for the whole observation period (119 children, 57 girls, mean age at T1D diagnosis: 7 years). A flowchart presents how the study group was reached and the reasons for dropout (**Figure 1**).

Diabetic ketoacidosis (DKA) was defined by blood pH <7.3 because of many missing records of bicarbonate and anion gap results. The definition of PR was based on ISPAD CPCG (insulin requirement <0.5 units/kg of body weight and HbA1c below 7%) (15). Insulin dose was assessed as daily insulin dose per kilogram of body weight (units/kg) (DIR).

This study focused on the observation of anthropometric data and remission presence throughout the 6-year period. Data collected at T1D diagnosis (gender, anthropometric data—weight, height, and BMI *Z*-score), laboratory test results (capillary blood pH, C-peptide concentration, positivity to anti-islet antibodies, presence of celiac and/or autoimmune thyroid disease) were analyzed in terms of their influence on the presence and duration of remission and were presented together with the values of all these parameters at the onset, in the previous publication (14). They are not presented or rediscussed in this study.

The studied population was divided into three subgroups according to PR duration: no PR at all (no partial remission—NPR), PR lasting less than 2 years (PR < 2), and PR at least 2 years (PR ≥ 2). These subgroups and their details were summarized in the flowchart (**Figure 1**).

The study was approved by the Ethics Committee of the Medical University of Silesia (PCN/0022/KB/175/20), and all procedures were conducted according to the Declaration of Helsinki.

## 3 Statistical analysis

The descriptive statistics (mean, standard deviation, median, lower and upper quartiles, interquartile range, skewness, and kurtosis) and their 95% confidence intervals were calculated for each continuous variable per patient group and time point. The categorical variables were described by their interval estimates of proportions and entropy-based relative diversity index. The comparative statistical analysis among groups and time points was performed with the use of the repeated measures ANOVA algorithm (rANOVA). The Lilliefors test was used for normality evaluation, while Mauchly’s test for sphericity was applied to verify the hypothesis on the homogeneity of variances. The Greenhouse–Geisser (GG) adjustment was chosen to correct for violation of sphericity if needed. Spearman correlation coefficient supported by the Jonckheere–Terpstra (JT) test on trend for the increase was applied to model the time response in the patient subgroups. Friedmann ANOVA test (fANOVA) was used to evaluate the significance of time response, and Kendall’s *W* concordance coefficient was calculated as the effect size measure. Wilcoxon test, together with Bonferroni correction for multiple testing, was performed in comprehensive cross-time comparative analyses. At each time point, the across-subgroups statistical tests were done for each variable of interest. Depending on the results of normality testing and Bartlett’s test for homogeneity of variances, one-way ANOVA (ANOVA) or non-parametric Kruskal–Wallis ANOVA (kwANOVA) was used, followed by the appropriate *post-hoc* tests. The *η* ^2^ was used as the measure of effect size for ANOVA analyses, and Glass Δ or rank biserial correlation coefficient (rbc) was used for the *post-hoc* tests.

## 4 Results

An overview of the basic statistics collected throughout the study is presented in **Table 1**, included in the supplement data (Appendix 1).

**Table 1:** Body mass index (BMI) *Z*-score at individual time points after type 1 diabetes onset.

	At diagnosis		After 2 years		After 4 years		After 6 years	
	N	%	N	%	N	%	N	%
***Z*-score < −2.00**	13	10.9	1	0.8	1	0.8	0	0.0
**−2.00 ≤ *Z*-score < −1.00**	20	16.8	11	9.2	12	10.1	6	5.0
**−1.00 ≤ *Z*-score < 1.00**	63	52.9	84	70.6	74	62.2	94	79.0
**1.00 ≤ *Z*-score ≤ 2.00**	19	16.0	19	16.0	28	23.5	15	12.6
***Z*-score >2.00**	4	3.4	4	3.4	4	3.4	4	3.4

The studied patients were classified by BMI *Z*-score using the distribution of the WHO reference population (18, 19) as follows: normal (BMI *Z*-score ≥ −1,00 and <1.00), overweight (BMI *Z*-score ≤2.00 and ≥1.00), and obese (BMI *Z*-score >2.00). There were 19 overweight (16%) and 4 obese (3%) children. **Table 1** presents the BMI *Z*-scores at individual time points after T1D onset.

During the whole observation period, PR was observed in 75 patients (63%), including 78.9% of overweight (17/19) and all of the obese patients (4/4). In all children that experienced PR, it started within the first 2 years after T1D diagnosis, mostly after 2.6 months. The mean duration of PR was 12.7 months, with 12 patients experiencing a PR longer than 2 years. Four of these patients had PR longer than 4 years, and for one patient, PR lasted for the whole observation time. No cases of complete remission were noted.

### 4.1 BMI *Z*-score according to remission presence and duration throughout the observation period

**Figure 2:**
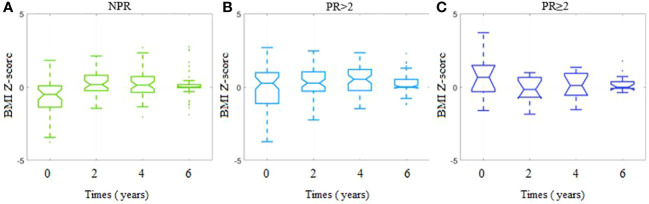
BMI Z-scores for each timepoint per study group depending on the presence and duration of partial remission. **(A)** Group with no partial remission (NPR). **(B)** Group with partial remission lasting less than 2 years (PR < 2). **(C)** Group with partial remission lasting at least 2 years (PR ≥ 2).

We compared the BMI *Z*-score values at chosen time points in separate subgroups in terms of PR (**Supplementary Figure 1**). The three remission-related groups differ significantly not only in the time response pattern but in the initial values of BMI *Z*-score (kwANOVA *p* = 0.008, effect size *η* ^2 ^= 0.08). The lowest mean initial BMI *Z*-score was observed among the NPR group (−0.65 ± 1.29), which increased to 0.02 ± 1.42 (*p* = 0.01, small effect rbc = 0.24) among the PR <2 group and remained similar to that level among patients from the PR ≥2 group (0.64 ± 1.43, *p* = 0.17, rbc = 0.12). We also analyzed the BMI *Z*-score value trends for all patients (**Supplementary Figure 2**) as well as for the PR subgroups (**Figure 2**). While the subsequent time points 2, 4, and 6 years are considered, the initial dissimilarity across patient groups declines (kwANOVA *p* = 0.35, *p* = 0.13, and *p* = 0.45, respectively) (**Supplementary Figure 2**). Within the NPR group, a significant increasing trend in BMI *Z*-score was observed (JT test *p* = 0.005), confirmed by fANOVA (*p* < 0.001) and Kendall’s coefficient of concordance (*W* = 0.20). The initial mean BMI *Z*-score significantly increased from −0.65 ± 1.29 to 0.20 ± 0.83 within the first 2 years (unadjusted *p* < 0.001) and remained at the same level till the end of the observations (**Figure 2A**). A different trend was observed for patients with partial remission (PR < 2): no consistent increase or decrease was observed (JT *p* = 0.40). The initial mean BMI *Z*-score was 0.02 ± 1.42, after 2 years 0.30 ± 0.98 and slightly increased to 0.50 ± 0.97 within the first 4 years of observation (*p* < 0.001), and then decreased to 0.23 ± 0.68 within the next 2 years (*p* = 0.04) (**Figure 2B**). In the last group (PR ≥ 2), similar to the NPR group, an observation was done. There is no significant trend in BMI *Z*-score in time among these patients (JT *p* = 0.31). The initial mean BMI *Z*-score of 0.64 ± 1.43 decreased within the first 2 years to −0.15 ± 0.95 (*p* = 0.02) and then increased significantly to the level of 0.07 ± 0.98 (*p* = 0.03) and remained unchanged for the last 2 years (**Figure 2C**).

### 4.2 HbA1c for individual subgroups in terms of PR in the studied time points

We compared the HbA1c values at the defined time points in the PR subgroups (**Supplementary Figure 3**). The patient groups did not differ in the mean initial HbA1c (NPR 12.17 ± 2.97 vs. PR < 2 11.30 ± 2.01 vs. PR ≥ 2 12.36 ± 1.26, kwANOVA *p* = 0.08, *η* ^2 ^= 0.04) (**Supplementary Figure 3A**). The ANOVA comparisons among the patient groups showed medium or less significant differences 2 years after (kwANOVA *p* = 0.006, *η* ^2 ^= 0.09), 4 years after (*p* = 0.002, *η* ^2 ^= 0.10), and 6 years after (*p* = 0.044, *η* ^2 ^= 0.05). The strongest and the most consistent descent in mean HbA1c was noticed in PR ≥2, with the final mean HbA1c equal to 6.98 ± 0.82, in comparison to NPR (final mean HbA1c 8.15 ± 1.87, *p* = 0.014, rbc = −0.33). It was expected taking into consideration the definition of the remission phase. PR <2, with its final mean HbA1c 7.80 ± 1.84, did not differ significantly from NPR (*p* = 0.188, rbc = −0.13) and PR ≥2 (*p* = 0.095, rbc = −0.20) (**Supplementary Figures 3B–D**).

### 4.3 DIR for individual subgroups in terms of PR in the studied time points

We compared the DIR at the defined time points in the PR subgroups. DIR values significantly differ in their time profiles across the patient groups (GG-corrected rANOVA *p* < 0.001). No significant trend in time was observed in NPR (JT *p* = 0.489, fANOVA *p* = 0.299, *W* = 0.03), with a slight increase in time noticed for PR <2 (JT *p* = 0.001, fANOVA *p* < 0.008, *W* = 0.08) and significant growth in PR ≥2 (JT *p* < 0.001, fANOVA *p* < 0.001, *W* = 0.65). As expected, the mean DIR differs significantly with a large effect among groups at 2 years after (ANOVA *p* < 0.001, *η* ^2 ^= 0.32), with the effect deteriorating in time (*η* ^2 ^= 0.09 at 4 years and 0.02 at 6 years). The final mean DIRs do not differ among the patient groups 6 years after (kwANOVA *p* = 0.272).

## 5 Discussion

During the 6 years of follow-up, 63% of the children in the study group experienced PR. The prevalence of PR is in line with previous reports describing PR in 56%–70% of patients (depending on the accepted definition of PR), developing typically within the first 2 years of T1D diagnosis (4, 20–22). Also, in our study population, in all cases, PR started in the first 2 years after T1D onset; usually, PR began during the first 3 months of observation.

Like other authors, we noted a gradual decrease in the number of remitters with time (2, 4, 6, 7, 21, 23, 24). Merely 10% (12 patients) of children still had PR after 2 years from diagnosis, and in only a few cases, PR lasted more than 4 years, and we observed only one case with PR prolonged over 6 years. Among the studies regarding PR, one study conducted in Germany and Austria observed 3,657 children with T1D over an equally long period of 72 months (7). The authors reported that most PR occurred during the first 3 months after T1D clinical manifestation and lasted 9 months on average. They also observed a prolonged PR: 5 years in 7% of the patients and 6 years in 5% of the patients (7).

Among the studied population, we did not find any case of complete remission, which is consistent with some previous studies (2, 4, 21). However, some other authors reported complete remission cases (10, 23, 25).

The analysis revealed that almost one-fifth of our investigated cohort were children who were overweight and obese at T1D diagnosis—frequencies only slightly higher than reported in other studies (18% and 11%) (4, 26). Importantly, throughout the entire observation period, we noted a decrease in the number of overweight patients—by approximately 3% after 6 years. During the same time, the rate of obesity stayed at the same level, but it needs to be noted that these were not the same patients that were obese at T1D onset. This trend is also shared with other publications. In a Spanish longitudinal study, the prevalence of obesity remained constant at close to 6% and even slightly increased (6.7%) after 9 years from T1D onset (27). A 10-year follow-up from the United States showed a much higher percentage of obese and overweight patients (33%) that did not change significantly throughout the duration of the study despite intensive treatment (28). In contrast, authors from Italy reported that during 6 years from T1D onset, the rate of overweight and obese patients increased, respectively, from 7.9% to 15.6% and from 3% to 6.5% (29).

In general, we observed a gradual normalization of the BMI *Z*-score, as pictured in **Figure 1**, included in the **Supplement Data**. This is particularly surprising, considering that over the course of the study, most patients entered puberty, which is naturally associated with weight gain. Our patients, despite the disease and the need for continuous insulin therapy, managed to normalize their weight. Many factors could have contributed to this positive phenomenon, such as a greater awareness of T1D and related health issues, knowledge about possible complications which may develop in adulthood, regular diabetes and nutritional education along with psychological support, regular medical checkups, and finally, the emotional growth of patients. The gradual shift toward the normalization of BMI *Z*-score seems to be important because it was shown that overweight and obese children with T1D had a higher prevalence rate of hypertension, metabolic syndrome, and high alanine aminotransferase compared with normal-weight children with T1D (30).

However, it should be mentioned that after the first 2 years, normalization of BMI *Z*-score was seen mostly in patients without PR, and during the following years, they managed to keep the BMI *Z*-score constant. On the contrary, patients with PR kept their BMI *Z*-score constant after 2 years, and at the next time point, when the protective honeymoon period ended, their BMI *Z*-score was much higher. This observation may be explained by the fact that patients with PR were less likely to follow medical recommendations and impose a strict healthy lifestyle during the PR. However, when the PR ended, this behavior became noticeable in the BMI *Z*-score. Hopefully, the BMI *Z*-score normalized after 6 years which could result from a correction of bad habits, thanks to an education with an emphasis on proper nutrition and a reduction of sedentary lifestyle.

Higher BMI *Z*-score values among remitters compared with children with T1D not experiencing PR were reported and discussed in the previous article (4). Within the studied cohort, PR was more common in overweight and occurred in all obese patients. This study revealed that this difference in BMI *Z*-score was not sustained 2, 4, and 6 years after T1D onset.

Previous studies noted that there is no difference in HbA1c at diagnosis at T1D onset between remitters and non-remitters (4, 6, 20, 22–24). After 2 years of follow-up, the level of HbA1c for all patients with PR was significantly lower compared with non-remitters, which is not surprising considering the definition of PR and as observed before (22). Data from this study (22) confirmed, like ours, that DIR for remitters is significantly lower after 2 years from diagnosis, but after 4 years, this effect is visible only for patients with remission longer than 2 years, and after 6 years of follow-up, DIR is comparable for all patients. Nwosu et al. reported that after 4–5 years of observation, there was no statistical difference in DIR between remitters and non-remitters (*p* = 0.24) (12). At the beginning of the disease, a higher BMI is associated with insulin resistance resulting from being overweight or obese. A healthy lifestyle leading to a reduction of body weight contributed to the decrease in insulin requirement. However, the β-cell destruction process progresses, and despite the normalization of body weight and proper insulin sensitivity, the PR ends and DIR increases.

The current study has several limitations that should be taken into account in the interpretation of the results. First of all, the data were analyzed from a single center in the same geographic region. Due to the retrospective analysis, we did not have sufficient data on C-peptide levels, which are often described to be related to BMI, especially among obese patients (26, 31). Another limitation is its retrospective design, which limits the possibility of assessing other important factors such as the method of insulin delivery, type of insulin intake, pubertal or family status, eating disorders, mental illness, smoking, or physical activity, which may also have a significant impact on BMI fluctuance (3, 32–34).

Nonetheless, our results are based on a long-term follow-up of the same large group of patients. Patients were followed up by the same diabetes team, received similar diabetes education, and were given the same strict goals according to the yearly published recommendation of Diabetes Poland from 2005, such as intensive intravenous insulin therapy in the first 1–2 days of hospitalization, followed by subcutaneous insulin therapy with adjustment of the appropriate insulin dose to maintain normoglycemia. The representative sample of remitters and non-remitters is an additional advantage that gave the ability to compare the differences between the groups.

## 6 Conclusion

During 6 years of follow-up, PR occurred in almost two-thirds of the studied children including almost all overweight and obese children. The highest rate of PR was observed during the first 3 months and no cases of PR starting 2 years after diabetes onset. Remitters had higher BMI *Z*-scores at T1D onset. We observed a gradual normalization of the BMI *Z*-score at the end of the follow-up. In the NPR group, the mean BMI *Z*-score remained constant after an initial increase. In both remitter groups, the increase in BMI *Z*-score appeared later when the protective honeymoon period ended. Regardless of BMI *Z*-score, the β-cell destruction process progresses, and after 6 years, the DIR is similar for all patients.

## Data availability statement

The raw data supporting the conclusions of this article will be made available by the corresponding author upon reasonable request.

## Ethics statement

The study was approved by the Ethics Committee of the Medical University of Silesia (PCN/0022/KB/175/20), and all procedures were conducted according to the Declaration of Helsinki. The studies were conducted in accordance with the local legislation and institutional requirements. Written informed consent for participation was not required from the participants or the participants’ legal guardians/next of kin in accordance with the national legislation and institutional requirements.

## Author contributions

MS-G: Data curation, Investigation, Methodology, Validation, Writing – original draft. PJ-C: Conceptualization, Funding acquisition, Supervision, Validation, Writing – review & editing. JP: Data curation, Formal Analysis, Validation, Writing – review & editing. AK: Data curation, Validation, Writing – review & editing. AC: Conceptualization, Funding acquisition, Investigation, Methodology, Supervision, Validation, Writing – review & editing, Writing – original draft.
