# Supplementary material for: Body mass index and partial remission in 119 children with type 1 diabetes—a 6-year observational study
Source: Front Endocrinol (Lausanne). 2023 Sep 14;14:1257758. doi: 10.3389/fendo.2023.1257758 (PMC10538636; doi:10.3389/fendo.2023.1257758)
Supplement: Supplementary file 1 [file Table_1.docx]

**Appendix 1.**

*Table 1. Basic statistics data for BMI Z-score, HbA1c and DIR at individual study time points.*

|  | | **BMI Z-score** | | **HbA1c** | | **DIR** | |
| --- | --- | --- | --- | --- | --- | --- | --- |
|  |  | M | SD | M | SD | *M* | *SD* |
| **At diagnosis of diabetes** | NPR | -0.65 | 1.29 | 12.17 | 2.97 | *-* | |
|  | PR<2 | 0.02 | 1.42 | 11.30 | 2.01 |  |  |
|  | PR≥2 | 0.64 | 1.43 | 12.36 | 1.26 |  |  |
| **After 2 years** | NPR | 0.20 | 0.83 | 7.30 | 1.34 | 0.84 | 0.16 |
|  | PR<2 | 0.31 | 0.98 | 7.20 | 1.28 | 0.73 | 0.21 |
|  | PR≥2 | -0.15 | 0.95 | 6.47 | 0.40 | 0.42 | 0.07 |
| **After 4 years** | NPR | 0.19 | 0.97 | 8.03 | 1.87 | 0.87 | 0.19 |
|  | PR<2 | 0.50 | 0.97 | 7.33 | 1.16 | 0.82 | 0.20 |
|  | PR≥2 | 0.07 | 0.98 | 6.68 | 0.63 | 0.64 | 0.22 |
| **After 6 years** | NPR | 0.13 | 0.96 | 8.15 | 1.87 | 0.85 | 0.16 |
|  | PR<2 | 0.23 | 0.68 | 7.80 | 1.84 | 0.83 | 0.16 |
|  | PR≥2 | 0.24 | 0.64 | 6.98 | 0.82 | 0.76 | 0.16 |

*M* – mean; *SD* – standard deviation
