# Supplementary figures and images for: Body mass index and partial remission in 119 children with type 1 diabetes—a 6-year observational study
Source: Front Endocrinol (Lausanne). 2023 Sep 14;14:1257758. doi: 10.3389/fendo.2023.1257758 (PMC10538636; doi:10.3389/fendo.2023.1257758)

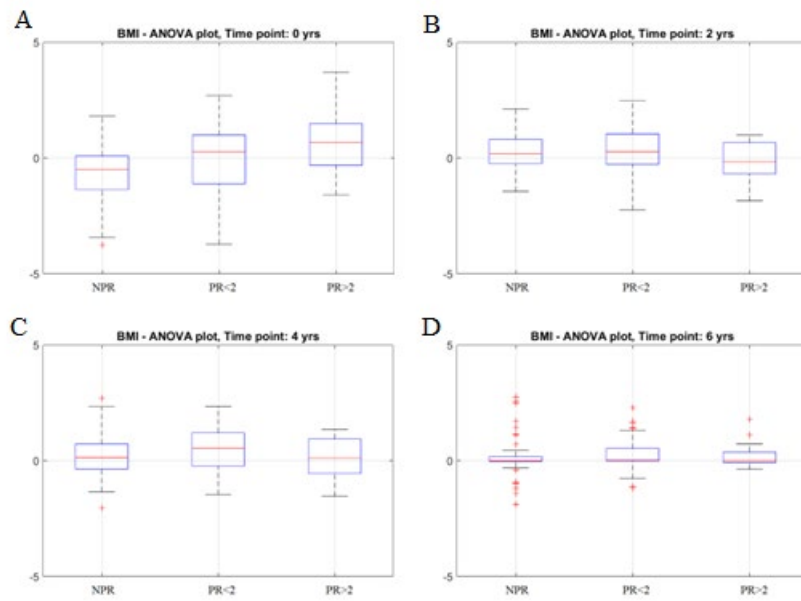

NPR - no partial remission, PR<2 - PR lasting less than 2 years PR $\geq$ 2 - PR longer than 2 years

Supplement: Supplementary file 2 [file Image_1.pdf]

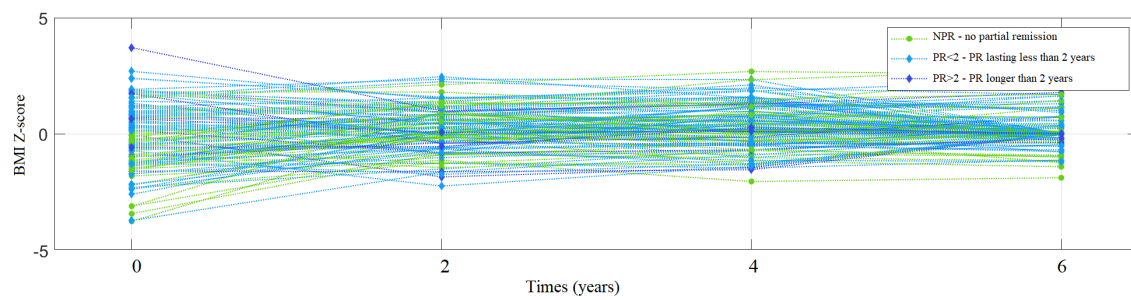

Supplement: Supplementary file 3 [file Image_2.pdf]

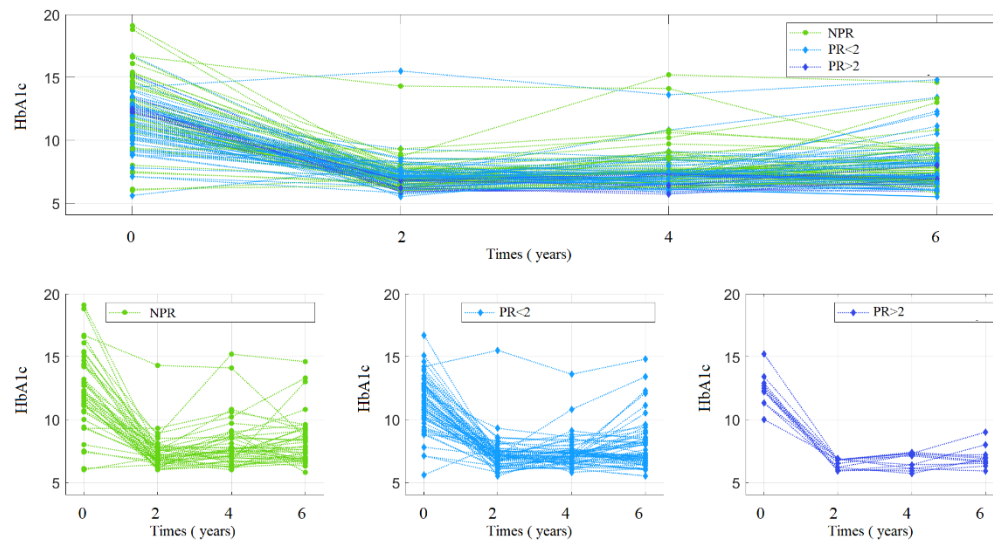

NPR - no partial remission, PR<2 - PR lasting less than 2 years PR≥2 - PR longer than 2 years

Supplement: Supplementary file 4 [file Image_3.pdf]
